# Imaging-based optical barcoding for relative humidity sensing based on meta-tip

**DOI:** 10.1515/nanoph-2021-0529

**Published:** 2021-11-02

**Authors:** Yin Liu, Xiaowei Li, Yufeng Chen, Guangzhou Geng, Junjie Li, Yongtian Wang, Lingling Huang

**Affiliations:** Beijing Engineering Research Center of Mixed Reality and Advanced Display, School of Optics and Photonics, Beijing Institute of Technology, Beijing 100081, China; Laser Micro/Nano-Fabrication Laboratory, School of Mechanical Engineering, Beijing Institute of Technology, Beijing 100081, China; Beijing National Laboratory for Condensed Matter Physics, Institute of Physics, Chinese Academy of Sciences, Beijing 100190, China

**Keywords:** dynamic time warping, fiber-optic humidity sensor, graphene oxide, hybrid metasurface, surface plasmon resonance

## Abstract

In a wide range of applications such as healthcare treatment, environmental monitoring, food processing and storage, and semiconductor chip manufacturing, relative humidity (RH) sensing is required. However, traditional fiber-optic humidity sensors face the challenges of miniaturization and indirectly obtaining humidity values. Here, we propose and demonstrate an optical barcode technique by cooperating with RH meta-tip, which can predict the humidity values directly. Such RH meta-tip is composed of fiber-optic sensor based on surface plasmon resonance (SPR) effect and graphene oxide film as humidity sensitizer. While SPR sensor is composed of multimode fiber (MMF) integrated with metallic metasurface. Dynamic time warping (DTW) algorithm is used to obtain the warp path distance (WPD) sequence between the measured reflection spectrum and the spectra of the precalibrated database. The distance sequence is transformed into a pseudo-color barcode, and the humidity value is corresponded to the lowest distance, which can be read by human eyes. The RH measurement depends on the collective changes of the reflection spectrum rather than tracking a single specific resonance peak/dip. This work can open up new doors to the development of a humidity sensor with direct RH recognition by human eyes.

## 1 Introduction

Humidity plays a vital role in many physical, chemical, and biological processes [1], [2], [3], [4], [5], [6]. Continuous monitoring and control of humidity in the environment are essential to control COVID-19’s transportation in aerosol [7], [8], [9]. A large number of humidity sensing technologies and devices have been developed [10], [11], [12], [13]. However, most of them are flammable, explosive, and vulnerable to substantial electromagnetic interference. In particular, its considerable volume is difficult to meet the growing demand for sensor miniaturization. The fiber-optic humidity sensor has recently attracted extensive interest because of its small size, high sensitivity, anti electromagnetic interference, multiplexing, and remote controlling abilities [14], [15], [16], [17], [18], [19], [20]. The change of air refractive index (RI) caused by humidity is tiny [21], so it is difficult to measure the change of RI directly with fiber-optic sensors. To improve the RH sensitivity of fiber-optic sensors, various humidity-sensitive materials combined with fiber end or side have been developed. However, the functionalization of the fiber side wall with RH sensitizers is still an obstacle to the miniaturization, resulting in low spatial measurement resolution.

Although the fiber-optic RH sensors showed good sensitivity and resolution performance, there are still some limitations in the traditional demodulation method based on tracking a specific resonant peak/dip. First of all, the humidity value cannot be obtained directly from the reflection or transmission spectra. The relative change of resonant peak/dip characteristics caused by humidity variation to the original state is measured to reflect RH value. Therefore, it is impossible to obtain the value of humidity only from the spectrum without knowing the initial humidity. Secondly, the monitoring parameters of a specific resonant peak/dip may have nonlinear changes or inflexion points, especially in fiber-optic sensors based on multimode interference. Although this problem can be solved by increasing the number of resonance peaks/dips monitored, it significantly increases the difficulty and workload of spectrum demodulation and reduces the utilization of spectral resonance peaks/dips. Therefore, it is urgent to find a suitable sensing solution to achieve a small adequate sensing volume and direct humidity measurement.

Here, a humidity-sensitive meta-tip cooperated with optical barcode technique is proposed to meet the requirements of high miniaturization level and direct acquisition of RH value. Through the combination of metallic metasurface and MMF, local surface plasmon resonance (LSPR) in metal nanoplasmon structures can be excited under normal incidence. It makes SPR sensor have unique advantages in sensing size and integration. The optical barcode technique based on a DTW algorithm gives the humidity value according to the overall changes of those resonance dips, which can overcome the limitations of the traditional humidity sensing system based on single-formant tracking. Hence, we can directly read the RH value from the optical bar code obtained by processing the collective behavior of multiple resonance dips in the reflection spectrum. Such imaging-based barcodes with RH meta-tip may pave the way towards a versatile miniaturized visual humidity sensor.

## 2 Working principle of the optical barcode technique

**Figure 1:**
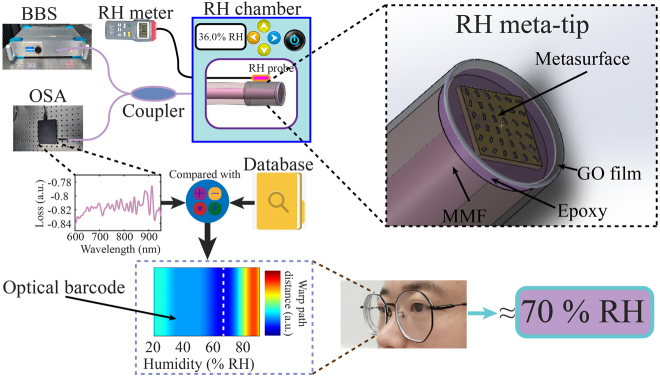
Schematic of RH measurement using the optical barcode technique.

As shown in Figure 1, the RH meta-tip reflection spectrum acquisition apparatuses are composed of broadband source (BBS), fiber coupler, and optical spectrum analyzer (OSA). A schematic diagram of RH meta-tip consists of SPR probe and GO film is shown in the inset. The SPR probe consists of MMF and metasurface with concentric-like phase profile. The metasurface is transferred to the end face of MMF by using thermosetting adhesive. The reflection spectrum of the RH meta-tip depends on the wavelength selective coupling between the hybrid modes of the SPR-fiber core and the guiding mode of the GO film. With the increase of the environmental humidity, GO film will have a swelling effect, which makes the thickness of the GO film increase and the RI decrease, and then the characteristics of these resonance dips are changed. The metasurface is composed of rectangular grooves with different rotation angles on the Au film [22, 23]. For the conventional SPR fiber-optic sensor with single humidity sensing capability, there is no need to phase modulate the incident light. In order to prove that the proposed optical barcode method does not rely on single-formant tracking, but on the collective behavior of spectral resonance peaks/dips, we use the metallic metasurface with concentric-like phase profile to phase modulate the incident light to produce a large number of resonance peaks/dips with different RI sensitivity. The design method of metasurface and working principle of the hybrid metasurface-GO film is described in detail in the supplementary materials (Figure S1).

Meanwhile, for RH direct detection, we develop optical barcode extraction process by considering that the reflection spectrum of the RH meta-tip has multiple resonance peaks/dips and some of them have inflection points in RH measurement. Such barcode sensing mechanism depends on the collective WPD between the measured reflection spectrum of the RH meta-tip prototype and the pre-calibrated spectral database. Firstly, the reflection spectra of multi equispaced humidity conditions are collected to form a database for precalibration. Then, the DTW algorithm is used to calculate those WPDs between the measured spectrum and the spectra of the database measured in advance. The WPD *DTW*(*m*,*n*) can be expressed as

$$\begin{array}{c} DTW\left(m,n\right)=\min\left[\sum_{k=1}^{\max\left(m,n\right)<K<m+n-1},D,P_{k}\right]\\ =d\left(Q_{m},C_{n}\right)+\min\left[DTW\left(m-1,n-1\right),\mathrm{(1)}DTW\left(m-1,n\right),DTW\left(m,n-1\right)\right]\text{ ,} \end{array}$$

where *m* and *n* are the number of elements of the measured sequence (*Q*) and the single sequence in the database (*C*). *DP*_*k*_ is the *k*th element of the contiguous set of matrix elements that defines a mapping between *Q* and *C*. *d*(*Q*_*m*_, *C*_*n*_) is the Euclidean distance between *Q*_*m*_ and *C*_*n*_. The resonance dips have different RH sensitivity and drift direction for the proposed RH meta-tip during the humidity change. In order to achieve better alignment, those spectral sequences need to be warped under the wavelength axis. The DTW algorithm is an effective way to realize spectral sequence regularization. The DTW algorithm can distinguishes the similarity between the measured spectrum and each element in the precalibrated database to obtain a warp path distance sequence. Afterward, the WPD sequence is transformed to the optical barcode, which can be used for direct RH detector. The value of WPD determines the color of the corresponding position of the optical barcode. Hence, the environmental humidity value can be preliminarily determined by the minimum WPD position using human eyes.

**Figure 2:**
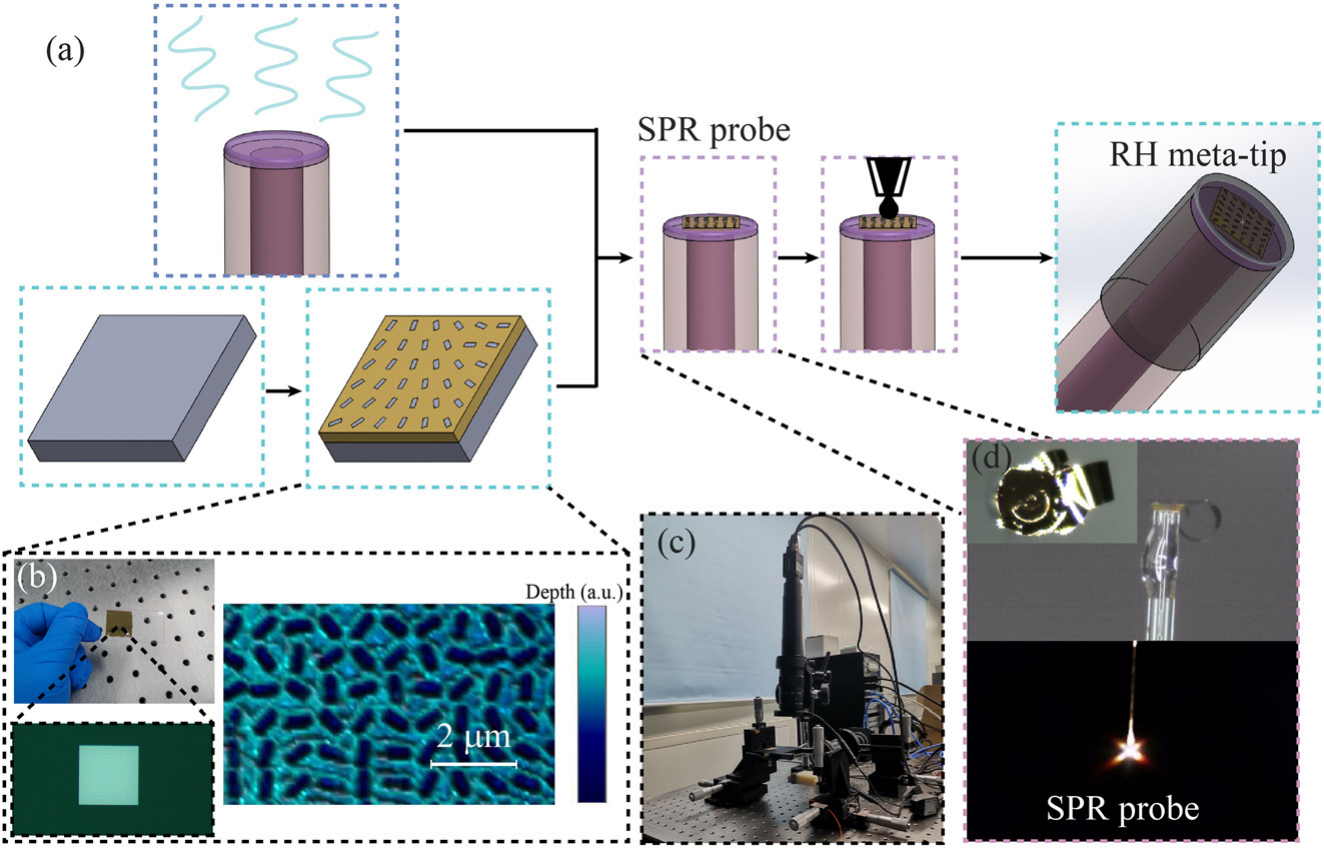
(a) Schematic diagram of the fabrication process of RH meta-tip. (b) Optical microscope image (left) and atomic force microscope (AFM) image (right) of the metallic metasurface. (c) Experimental system for MMF core and metasurface alignment. (d) Optical images of SPR probe tip.

The preparation process of the RH meta-tip is shown in Figure 2(a). Firstly, Au film was evaporated on one side of the quartz substrate. The thickness of the evaporated Au film is measured as 91.3 nm. Electron beam lithography (EBL) technology was used to prepare rectangular grooves with different rotation angles on the Au film to form the metasurface. The local features of the metasurface on the Au film can be seen in Figure 2(b). The thermosetting glue was dropped on the MMF end face and precured to improve the robustness of the metasurface embedded in the end face of the MMF by curing at room temperature. An optical microscope detection system was used to ensure that the center of the fiber end face and the metasurface coincide with each other, as shown in Figure 2(c). The optical image of the SPR probe tip and the photo of the far-field optical diffraction pattern is shown in Figure 2(d). Optical images and diffraction patterns indicate that the metasurface has been transferred to the end of the MMF. So far, the SPR probe has been prepared. In order to improve the humidity sensitivity of the SPR probe, it is necessary to prepare a GO film on the metasurface. The prepared SPR probe prototype was fixed on the glass slide, and GO dispersion with 4 mg/mL was dropped on the SPR probe and dried at room temperature for 4 h. After the solvent in the GO dispersion evaporated, the GO film could be covered on the SPR probe and become RH meta-tip. Reflection spectrum characteristics of SPR probe and properties of GO films can be seen in Figure S2 in the Supplementary Materials.

## 3 Experiment and discussion

The response of the RH meta-tip to the external humidity is attributed to the response of the SPR probe as well as GO film. Because the metasurface is composed of concentric phase profile, the reflection spectrum of the SPR probe prototype supports multiple modes of resonance dips due to the constructive and destructive resonances on metasurface (See Supplementary Materials). The RI response of those dips appearing in the reflection spectrum depends on the mode occupying the principal component, which will lead to the different resonance wavelength shift trends [24]. The change of the mode occupying the main component of the resonant dip lead to the wavelength and intensity of those dips changes dynamically, so the inflection point of the central wavelength changes with the environmental RI [25]. This phenomenon can make it challenging to obtain the humidity change by monitoring the central wavelength of the specific resonance dip. It can be seen from the Supplementary that it is difficult to obtain the humidity change by tracking the variation of the central wavelength of a single specific resonant dip over the RH range of 20–90% RH. In addition, the humidity value cannot be read directly.

In contrast, the optical barcode technology we developed does not rely on a single specific resonance dip, but on the collective behavior of multiple resonance peaks in the reflection spectrum. By measuring the similarity between the reflection spectrum of unknown RH value and the database spectrum, the bar code with the human eye recognizable humidity value is generated.

**Figure 3:**
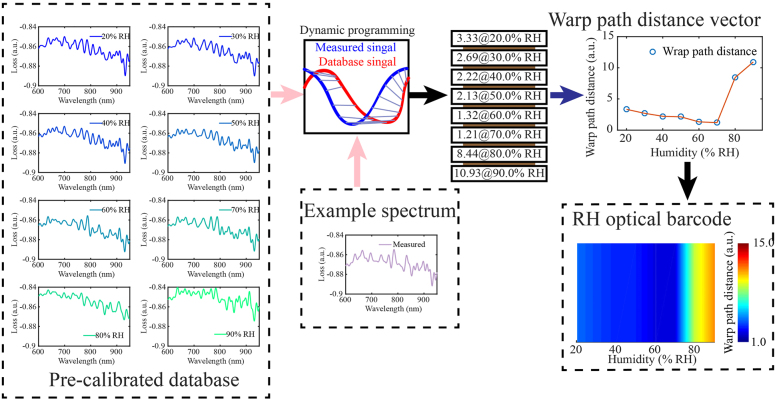
The process of extracting the RH optical barcode from the reflection spectrum of the RH meta-tip.

To understand the technical route, the workflow of the optical barcode method is shown in Figure 3, and the measured reflection spectrum at environmental humidity of 67.8% RH is taken as an example. The database consists of eight preacquired reflectance spectra of the RH meta-tip in the 20–90% RH range with a step of 10% RH. All the reflection spectra are measured in the wavelength range of 600–950 nm by using spectrometer. According to the feature information provided by the reflection spectrum of the RH meta-tip prototype, we use the WPD to represent the similarity between the measured spectrum and the database spectral elements. The smaller the WPD value, the higher the similarity between the two spectra. The calculation method of WPD is shown in Eq. (1). Then we get eight WPD values as one-dimensional array elements in order. We convert this one-dimensional array into an optical barcode. The size of each element in the one-dimensional array determines the color of the corresponding barcode area. The human eye search for the color region representing the lowest value of WPD from the optical barcode, and the humidity value corresponding to the color region is the humidity value corresponding to the measured spectrum. From the barcode image, we can see that the actual humidity of the example spectrum is between 60% RH and 70% RH. From the overall situation of the color, the actual humidity is closer to 70% RH than 60% RH, which is consistent with the humidity measured by the hygrometer@67.8% RH. The generated barcode provides an intuitive method for data visualization and is easy to identify. By changing the colormap of WPD, one can further increase the sharpness and contrast of barcode and improve the friendliness of human eye recognition. The value of humidity and optical barcode can intuitively transmit the RH information to human beings. The humidity value obtained from the WPD vector is discrete and finite. Optical barcode can be used to judge which humidity range the ambient humidity is more inclined to, which is more friendly than the humidity value.

**Figure 4:**
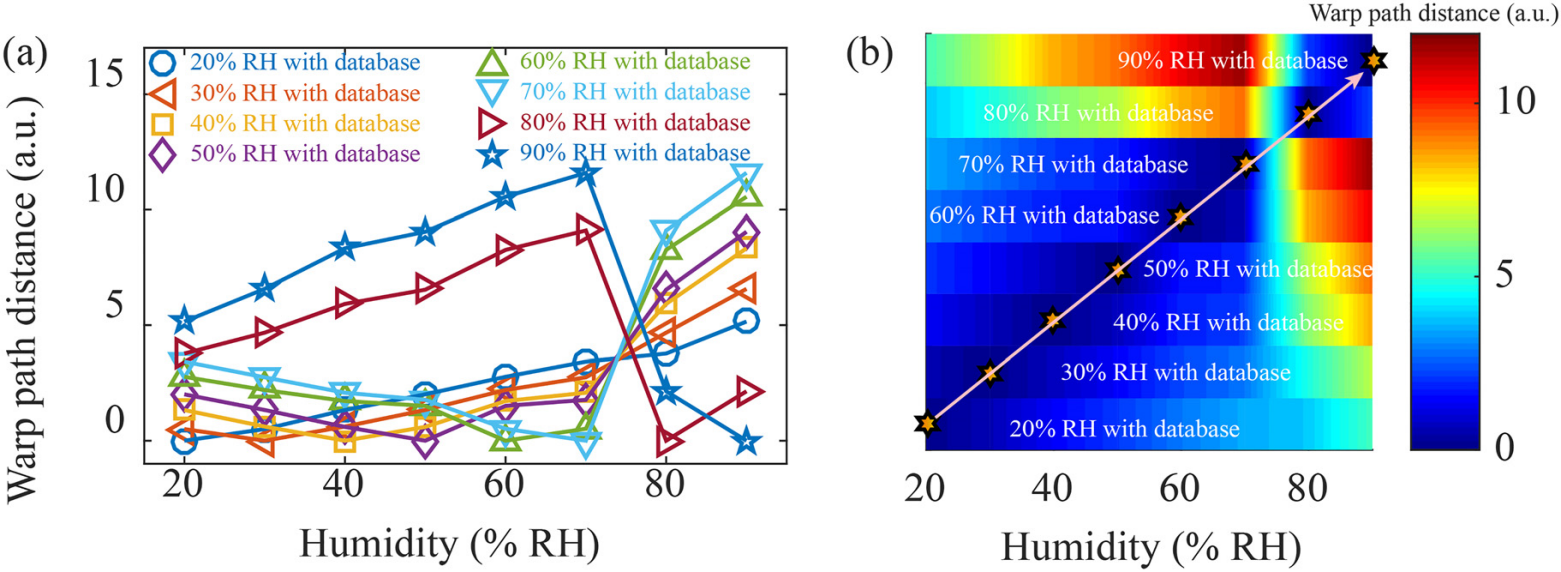
(a) WPD from an internal comparison of the precalibrated database. (b) Corresponding barcodes of (a).

To further verify the effectiveness of this optical barcode generation method, each element in the database corresponding to different RH are treated as the example spectrum to process by the DTW algorithm with the database. Figure 4(a) shows the resulting WPD sequences corresponding to different humidity conditions, that is, each WPD sequence is obtained by processing the corresponding single database element with the whole database. The WPD sequences appear slightly abnormal near 70–80% RH because there are two swelling coefficients in GO film. In less than 80% RH environment, the expansion of GO film is mainly caused by the H-bonding between water molecules and the carboxylic group. In more than 85% RH, the expansion of GO film is mainly caused by the H-bonding between water molecules [26, 27]. The barcodes corresponding to Figure 4(a) are arranged in order of humidity, as can be seen in Figure 4(b). The hexagon represents the approximate lowest position of WPD sequence under different humidity conditions, that is, the actual humidity value of the example spectrum. It can be seen from Figure 4(b) that the actual humidity of the RH meta-tip can be approximately obtained by barcode over the entire humidity measurement range.

**Figure 5:**
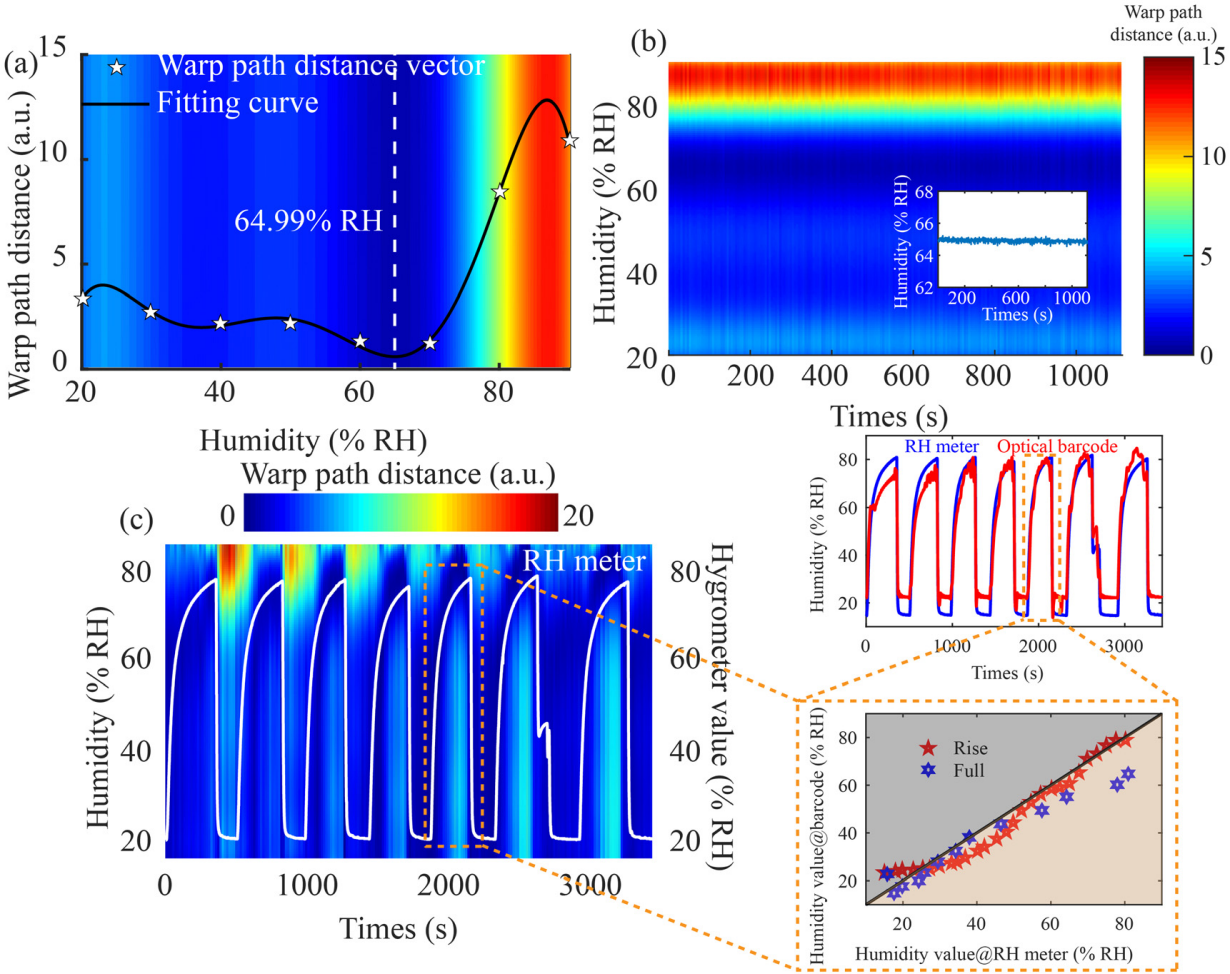
(a) RH optical barcode@67.8% RH and fitting curve of WPD sequence. (b) A stability test result of the RH optical barcode@67.80% RH. (c) Characterization of reversibility and repeatability of the RH optical barcode.

As can be seen from Figure 3, by judging the minimum value of the WPD sequence responding to the RH value through the barcode, the humidity value obtained by human eyes is only an approximate estimate of the ambient humidity. Because the humidity value corresponding to the pre-calibrated spectral database is discrete, and the humidity interval is limited by the accuracy of the hygrometer and RH chamber, it is impossible to improve the position discrimination accuracy of WPD by reducing the humidity interval of the spectral database. To improve the display resolution by reducing the uncertainty in determining the humidity position corresponding to minimum WPD, we use the high-order polynomial fitting method to extract the RH value corresponding the minimum WPD. The coefficients of the polynomials can be obtained [28]:

(2) $$\frac{\partial \left(\sum_{RH_{i}\in Dat}{\left[Dis\left(RH_{i}\right)-\int_{j=0}^{n}p_{j}RH_{i}^{j}\right]}^{2}\right)}{\partial p_{j}}=0\text{ ,}$$

where $RH_{i}$ is the element of the Dat , Dat is the collection of RH points, $Dis\left(RH_{i}\right)$ is the WPD sequence, $p_{j}$ is the coefficient defining of the polynomial, *n* is the polynomial degree. Here we choose 6 as the polynomial degree. The fitting curve of humidity and WPD is shown in Figure 5(a). The calculated humidity value is approximately 64.99% RH from the fitting results, while the measured value with the RH meter is 67.8% RH. This slightly deviation may be caused by measurement errors or few elements in the database. Increasing the number of reflection spectra in the precalibrated database may improve the measurement accuracy. After fitting WPD vector with high-order polynomial, humidity value, and optical barcode are very effective direct humidity acquisition methods. However, by refreshing the optical barcode, especially when the ambient humidity is at the edge of the precalibrated humidity range, the abnormal WPD vector can be identified according to the optical barcode, so as to eliminate the unreliable data and improve the robustness of humidity measurement. This is the advantage and necessity of using optical barcode instead of RH value.

Meanwhile, stability is an important parameter to characterize the performance of the fiber-optic RH sensor, so we measured the reflection spectra of the RH meta-tip prototype at 67.8% RH over 1100 s in the steps of 1 s to verify the stability, as shown in Figure 5(b). Those humidity values are calculated from the barcode as shown in the inset. It can be seen that the RH meta-tip has good stability. To further study the RH optical barcode’s dynamic performance, seven cycles of humidity switching are measured. The environmental humidity was set to switch between 15% RH and 80% RH. The database consists of reflectance spectra in the same range with RH step of 5%. The dynamic change of RH barcode and the ambient humidity value measured by the hygrometer are shown in Figure 5(c) as comparison, which shows that the RH optical barcode has good human eye distinguishing characteristics. The comparison between the humidity values obtained by fitting the WPD array and the hygrometer values is shown in the inset of Figure 5(c). In one cycle, the comparison between the measured values@RH meter and the experimental values@barcode in the ascending and descending processes is also demonstrated. The reason for the significant difference between the measured results and the experimental results at the edge@15% RH and 80% RH may be that the fitting and extremum processing is not good at processing the edge data, and the expansion coefficient of GO film at high levels is obviously different from that at low levels. Nevertheless, the fluctuation of power in the process of reflection spectrum acquisition will eventually cause the change of fitting results.

## 4 Conclusions

In summary, we have demonstrated an optical barcode technique combined with an RH meta-tip for RH direct reading. The experimental results showed that distinguishing WPD is suitable for calculating the spectral similarity of multiple resonance peaks with different sensitivities and drifting trends. The proposed RH meta-tip with tiny size is also suitable for gas monitoring, such as isopropanol, ammonia, formaldehyde, acetone, and ethanol, due to the nonselective property of GO film. Such optical barcode technique does not require additional complex design and expensive components, which can be combined with state-of-the-art surface functionalization technology for more complex chemical analysis or bioassays. The imaging-based technology has the potential to be used as a general technology in other types of optical resonant sensors with multi resonant peak/dip characteristics, such as wall gallery mode sensors, Mach–Zehnder interferometer, Michelson interferometer, and multimode interferometric sensors. The optical barcodes obtained by our proposed method provide unique possibilities for advanced image analysis and pave the way for multifunctional and miniaturized transmission/reflection spectroscopic devices.

## Supplementary Material

Supplementary Material
