# Supplementary material for: Imaging-based optical barcoding for relative humidity sensing based on meta-tip
Source: Nanophotonics. 2021 Nov 2;11(1):111–8. doi: 10.1515/nanoph-2021-0529 (PMC11501753; doi:10.1515/nanoph-2021-0529)
Supplement: Supplementary file 1 — Supplementary Material [file j_nanoph-2021-0529_suppl.docx]

**Imaging-based optical barcoding for relative humidity sensing based on meta-tip**

*Yin Liu*^1^, *Xiaowei Li*^2, *^, *Yufeng Chen*^1^, *Guangzhou Geng*^3^, *Junjie Li*^3^, *Yongtian Wang*^1^, *Lingling Huang*^1, *^

1.Beijing Engineering Research Center of Mixed Reality and Advanced Display, School of Optics and Photonics, Beijing Institute of Technology, Beijing 100081, China

2. Laser Micro/Nano-Fabrication Laboratory, School of Mechanical Engineering, Beijing Institute of Technology, Beijing 100081, China

3. Beijing National Laboratory for Condensed Matter Physica, Institute of Physics, Chinese Academy of Sciences, Beijing 100190, China

Corresponding authors: Xiaowei Li, [lixiaowei@bit.edu.cn](mailto:lixiaowei@bit.edu.cn), Lingling Huang, [huanglingling@bit.edu.cn](mailto:huanglingling@bit.edu.cn).

**1. Design method of the metasurface and working principle of the GO film**


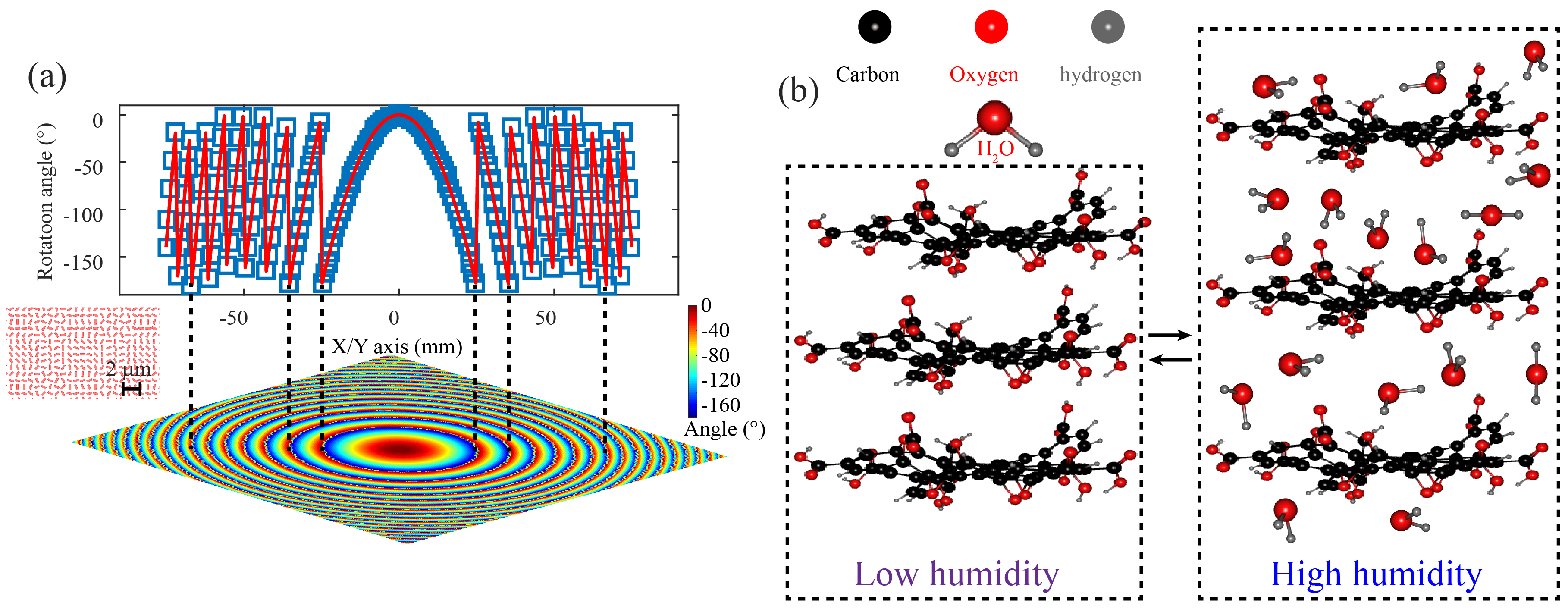


Figure S1 (a) Angle distribution of rectangular Au grooves on the metasurface. (b) Schematic of a GO film swollen by water molecules under the low RH and the high RH levels

The metasurface is composed of rectangular grooves with different rotation angles on the Au film,^[1]^ as shown in Figure 1(a). These structural parameters such as period, length, and width of the groove were set as 750 nm, 610 nm, and 220 nm, respectively. The rotation angles are arranged to$-K\text{π}\left(\sqrt{r^{2}+A^{2}}-A\right)$, where *r* is the distance between the geometric center of the metasurface and the center of the rectangular groove. K and A were set to set to 28.6 μm^-1^ and 150 μm , which are coefficients that determine the slope of the azimuth change, respectively. The metasurface generates resonant surface plasmon polaritons (SPPs) under incident light. The resonant light interacts with the GO sheets adsorbed on the metasurface, resulting in a change in resonance characteristics with humidity change. The SPR probe has a reflection spectrum in multiple resonance modes, with different field distributions on and within the metallic metasurface. So SPR probe has a specific RI response in each mode.

In high and low RH conditions, the swelling effect of GO film caused by infiltration of water molecules is shown in Figure 1(b). With the increase of humidity, more water molecules can be adsorbed in the nanopores of the GO films. The Fermi level of GO increases at the Dirac point, which leads to the blocking of interband transition and the decrease of conductivity.^[2, 3]^ When the environmental RH reaches a higher level, the H-bonding between the functional groups and the water molecules connecting the adjacent GO layers under low RH will be replaced by the H-bonding of the water molecules, resulting in the unneglectable swelling of the GO film.^[4]^

**2. Reflection spectrum characteristics of SPR probe, and properties of GO films**


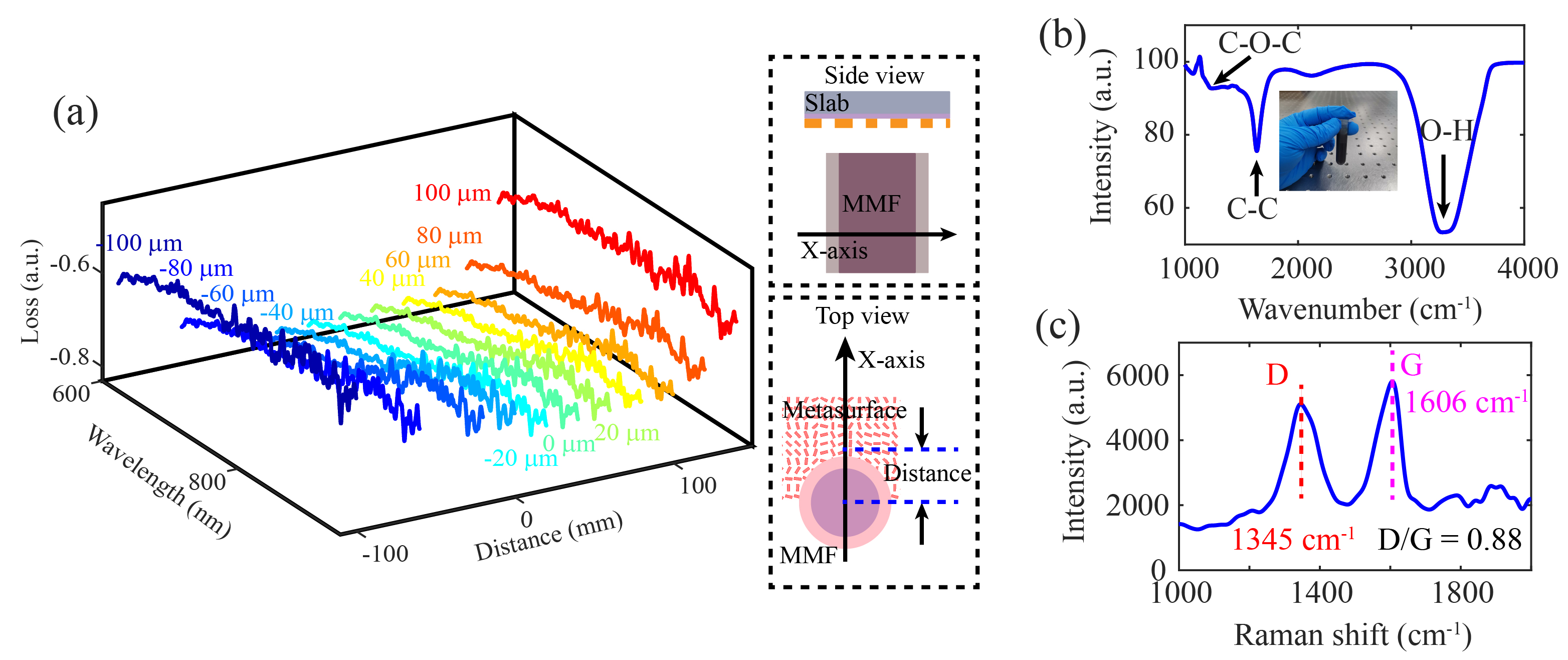


Figure S2 (a) Reflection spectra with the different central distances between MMF and metasurface. (b) FTIR spectrum and (c) Raman spectrum of the GO dispersion.

The reflection spectra at different distances between the geometric center of MMF and metasurface were investigated, as shown in Figure S2(a). To ensure consistency in the measurement process, the medium between the metasurface plane and the MMF end face is air, and the distance should be kept consistent. It can be seen from Figure S2(a) that the overall power of the reflection spectrum increases with the increasement of the geometric center distance. Because the localized SPR effect reduces when the overlap area between the MMF core and metasurface decrease. The reflection spectrum can be flexibly adjusted by controlling the superposition region.

The GO dispersion was tested by Fourier transform infrared (FTIR) absorption spectrum to prove the existence of hydrophilic groups in the GO film, as shown in Figure S2(b). The characteristic peaks (the O-H vibration@3297 cm^-1^, the C-O-C vibration@1224 cm^-1^, and the C-C vibration@1638 cm^-1^) in Figure 2(b) showed that the GO sheets contain a large number of hydrophilic functional groups. The Raman spectrum of GO dispersion is shown in Figure S2(c) to confirm the atomic structure of the used GO film. The relatively low D/G intensity ratio (0.88) once again proves that there are a large number of hydrophilic groups, disordered carbon, and defects in the GO atomic structure,^[5]^ which helps to enhance the strong adsorption of water molecules, thus enhancing the sensitivity of the meta-tip to humidity.

**3. RI response of the SPR probe and the RH response of the meta-tip**


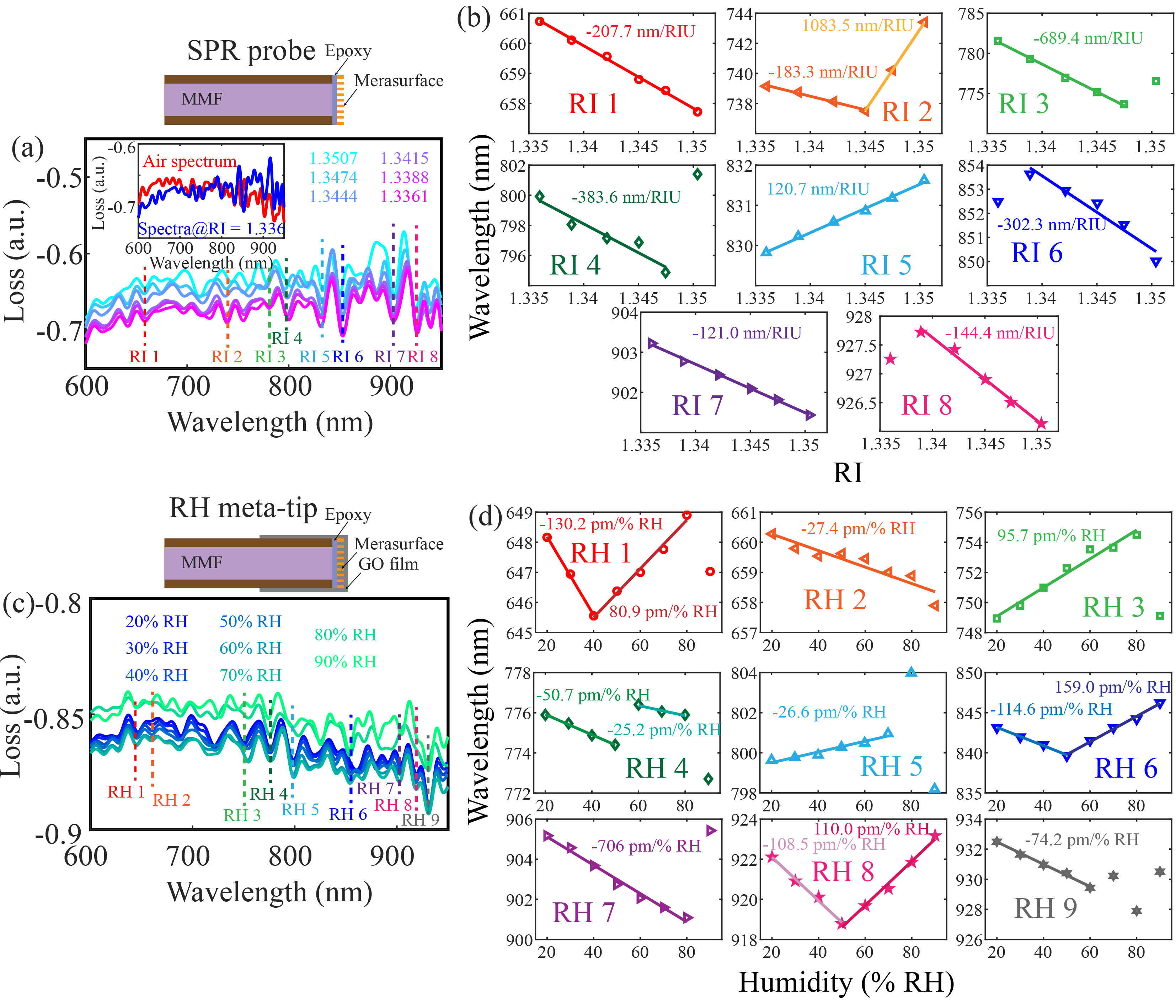


Figure S3 (a) Reflection spectra of the SPR probe under different RI. (b) The central wavelength of those resonance dips varies with RI. (c) Reflection spectra of the proposed RH meta-tip under different environmental humidity. (d) Relationships between the RH and the central wavelength of those dips.

We measured the RI response of the SPR probe without GO film first. The reflection spectrum of the SPR probe prototype is composed of multiple modes of resonance dips. The evolution of reflection spectra of the SPR sensing probe prototype with RI is shown in Figure S3(a). Those resonant dips with central wavelengths of about 658.5 nm, 738.9 nm, 781.2 nm, 800.4 nm, 831.1 nm, 852.9 nm, 902.5nm, and 926.6nm were selected as RI1, RI2, RI3, RI4, RI5, RI6, RI7, and RI8, respectively. The relationship between the central wavelength of different resonant dips and the RI is shown in Figure S3(b). It can be seen from Figure S3 (b) that the variation trends of those central wavelengths are quite different. Apart from RI2, all the other resonances have linear and monotonic trends within a specific range of RI. This is because, the change of the mode occupying the main component of the resonant dip lead to the wavelength and intensity of those dips changes dynamically, so the inflection point of the central wavelength changes with the environmental RI.^[6]^ This phenomenon can make it challenging to obtain the humidity change by monitoring a single specific resonance dip.

After the SPR probe prototype is coated with GO film, the evolution of the RH meta-tip reflection spectrum with humidity is shown in Figure S3(c). In Figures S3(a) and S3(c), the reflection spectra of the SPR probe prototype are different under different ambient RI,^[7]^ mainly because the principal component of the resonant peaks/dips may change, and the RI sensitivity of the resonant peak/dip depends on the RI sensitivity occupying the principal component.^[8]^ With the change of the external RI, the essential component of the resonant dip is also dynamically changing. The resonance dips with the central wavelength of approximately 645.1 nm, 661.4 nm, 753.7 nm, 775.8 nm, 798.7 nm, 840.0 nm, 903.5 nm, 919.1 nm, and 930.5 nm were labeled RH1, RH2, RH3, RH4, RH5, RH6, RH7, RH8, and RH9, respectively. The central wavelength of those resonant dips varies with humidity is shown in Figure S3(d). It can be seen from Figure S3(d) that the resonance dips RH1, RH6, and RH8 are linear in the humidity range on both sides of the inflection point. It is difficult to distinguish the RH trend from the relative displacement of the central wavelength of a single resonant dip to the original state for those dips RH1, RH6, and RH8. Those resonance dips of RH2, RH3, RH4, RH5, RH7, and RH9 are not monotonous and non-linear in the overall humidity measurement range, so it is difficult to get the humidity change in the overall humidity measurement range by using a single resonance dip according to the traditional humidity measurement method. It can only be spliced multiple effective humidity ranges by monitoring the wavelength of multiple dips. Hence, our method of optical barcode skillfully avoids the disadvantage of obtaining RH value by relying on the center wavelength of a single specific resonant peak/dip, and paves the way for direct identification of humidity.

**Reference**

[1] Chen X, Huang L, Mühlenbernd H, et al. Dual-polarity plasmonic metalens for visible light [J]. Nature Communications, 2012, 3: 1198.

[2] Schedin F, Geim A, Morozov S, et al. Detection of individual gas molecules adsorbed on graphene [J]. Nature materials, 2007, 6(9): 652.

[3] Li Z, Henriksen E A, Jiang Z, et al. Dirac charge dynamics in graphene by infrared spectroscopy [J]. Nature Physics, 2008, 4(7): 532.

[4] Medhekar N V, Ramasubramaniam A, Ruoff R S, et al. Hydrogen bond networks in graphene oxide composite paper: structure and mechanical properties [J]. ACS nano, 2010, 4(4): 2300-2306.

[5] Huang Y, Zhu W, Li Z, et al. High-performance fibre-optic humidity sensor based on a side-polished fibre wavelength selectively coupled with graphene oxide film [J]. Sensors and Actuators B: Chemical, 2018, 255: 57-69.

[6] Liu Y, Xia Q, Zhou A, et al. Multi-parameter sensing based on surface plasma resonance with tungsten disulfide sheets coated [J]. Optics Express, 2020, 28(5): 6084-6094.

[7] Cheng, Li, Xiyu, et al. Ultrafast miniature fiber-tip Fabry-Perot humidity sensor with thin graphene oxide diaphragm [J]. Optics Letters, 2018, 43(19): 4719-4722.

[8] Zhang Y, Zhou A, Qin B, et al. Refractive index sensing characteristics of single-mode fiber-based modal interferometers [J]. Journal of Lightwave Technology, 2014, 32(9): 1734-1740.
